# Correction for Kumar et al., “Escherichia coli Quorum-Sensing EDF, A Peptide Generated by Novel Multiple Distinct Mechanisms and Regulated by *trans*-Translation”

**DOI:** 10.1128/mBio.03436-20

**Published:** 2021-02-02

**Authors:** Sathish Kumar, Ilana Kolodkin-Gal, Oliver Vesper, Nawsad Alam, Ora Schueler-Furman, Isabella Moll, Hanna Engelberg-Kulka

**Affiliations:** a Department of Microbiology and Molecular Genetics, IMRIC, The Hebrew University-Hadassah Medical School, Ein Karem, Jerusalem, Israel; b Department of Molecular Genetics, Weizmann Institute of Science, Rehovot, Israel; c Max F. Perutz Laboratories, Center for Molecular Biology, Department of Microbiology, Immunobiology and Genetics, University of Vienna, Vienna, Austria

## AUTHOR CORRECTION

Volume 7, no. 1, e02034-15, 2016, https://doi.org/10.1128/mBio.02034-15. The positive-control complemented strain p*zwf* and negative-control mutant sample p*zwf*_701_AAA chromatogram were used multiple times in the figures in this manuscript only for clarity of the readers, since all the other sample peaks were compared with these two control samples. All experiments were performed with three consistent independent repeats.

